# Effects of high glucose conditions on the expansion and differentiation capabilities of mesenchymal stromal cells derived from rat endosteal niche

**DOI:** 10.1186/s12860-019-0235-y

**Published:** 2019-11-21

**Authors:** Ahmed Makki A. Al-Qarakhli, Norhayati Yusop, Rachel J. Waddington, Ryan Moseley

**Affiliations:** 1 School of Dentistry, Cardiff Institute of Tissue Engineering and Repair (CITER), College of Biomedical and Life Sciences, Cardiff University, Cardiff, CF14 4XY UK; 2 College of Dentistry, University of Anbar, Anbar, Iraq; 3 School of Dental Sciences, Universiti Sains Malaysia, Kelantan, Malaysia

**Keywords:** Mesenchymal stromal cells; Endosteum, Cellular senescence, Differentiation, Hyperglycaemia, Type II diabetes, Bone repair

## Abstract

**Background:**

Mesenchymal stromal cells in the endosteal niche lining compact bone (CB-MSCs) represent a heterogeneous population, all of which contribute to bone repair and remodelling. Hyperglycaemia associated with type 2 diabetes mellitus (T2DM) can delay and impair the bone healing process. Therefore, this study investigated the influences of high (25 mM) glucose conditions on CB-MSC populations isolated from male Wistar rats, versus normal (5.5 mM) glucose conditions; in terms of proliferation (population doublings, PDs), senescence characteristics, stem cell marker expression, colony forming efficiencies (CFEs); and osteogenic/adipogenic differentiation, following extended culture in vitro.

**Results:**

CB-MSCs under both normoglycaemic and hyperglycaemic conditions demonstrated similar morphologies and rapid exponential growth to >300PDs, although high glucose conditions promoted more rapid and persistent proliferation beyond ~50PDs, with few indications of senescence. Limited senescence was confirmed by minimal SA-β-galactosidase staining, low senescence marker (p53, p21^waf1^, p16^INK4a^) expression and positive telomere maintenance marker (rTERT, TR) expression. However, telomere lengths varied throughout culture expansion, with hyperglycaemia significantly reducing telomere lengths at PD50 and PD200. Furthermore, CB-MSCs expanded in normal and high glucose conditions remained non-transformed, exhibiting similar MSC (CD73/CD90/CD105), multipotency (CD146) and embryonic (Slug, Snail) markers throughout extended culture, but negligible hematopoietic (CD34/CD45) or pluripotency (Nanog, Oct4) markers. Hyperglycaemia significantly increased CFEs at PD50 and PD100, which decreased at PD200. CB-MSC osteogenic differentiation was also inhibited by hyperglycaemia at PD15, PD100 and PD200, but not at PD50. Hyperglycaemia inhibited CB-MSC adipogenic differentiation to a lesser extent at PD15 and PD50, with reduced adipogenesis overall at PD100 and PD200.

**Conclusion:**

This study demonstrates the limited negative impact of hyperglycaemia on the proliferative and stem cell characteristics of heterogeneous CB-MSC populations, although minor sub-population(s) appear more susceptible to these conditions leading to impaired osteogenic/adipogenic differentiation capabilities. Such findings potentially highlight the impact of hyperglycaemia on CB-MSC bone repair capabilities in situ.

## Background

Bone repair occurs via a multiple number of highly coordinated and overlapping events, involving various cell types, signalling molecules and extracellular matrix (ECM) components to re-establish normal bone architecture and function [1–4]. Such events are clinically important to successful fracture repair, orthopaedic/dental implant placement and bone augmentation outcomes. At the forefront of these reparative processes are the functions of mesenchymal stromal cells (MSCs), attributed to their defined self-renewal properties and abilities to undergo growth factor-driven, differentiation along the osteogenic lineage into mature osteoblasts; which drive ECM synthesis, deposition and mineralisation [1–3].

The primary sources of MSCs during bone repair have been suggested to exist within two principle, but distinct, niches within the bone marrow cavity [5]. The highly vascularised perivascular/sub-endosteal niche contains endothelial cells, hematopoietic stem cells (HSCs) and uncommitted MSCs [6–8]. In contrast, the endosteal niche exists at the interface between trabecular bone and bone marrow, organised around hematopoietic cells and uncommitted MSCs interacting with pre-osteoblasts and osteoblasts lining the compact bone [9, 10]. These more committed, lineage restricted bone lining progenitor cells have been proposed to represent the first responder cells during mineralised tissue repair and remodelling, with the immature MSCs subsequently proliferating to replace the former cell population [11, 12]. Recent characterisation studies on MSCs isolated from the endosteal niche of compact bone (CB-MSCs) have identified the ability to culture the pre-osteoblasts and osteoblasts along with immature MSCs to 15 population doublings (PD15), correlating with high osteogenic capabilities and minimal evidence of cellular senescence [12]. More prolonged culture expansion resulted in the more highly proliferative immature MSCs and transit amplifying (TA) cells dominating the cell population, which is commensurate with increased bi-potentiality for osteogenic and adipogenic induction. Notably these cells were able to continue to proliferate to beyond PD100 without exhibiting any classical characteristics of cellular senescence [12].

MSCs within both the perivascular and endosteal niches possess important roles in facilitating bone repair processes overall [1, 5]. However, despite bone healing being a highly organised process, it is recognised that these mechanisms can be significantly altered and/or impaired by the local tissue microenvironment, including by metabolic, cellular and molecular changes induced through the uncontrolled glycaemic control and hyperglycaemia associated with type 2 diabetes mellitus (T2DM), initiated as a consequence of insulin resistance [13, 14]. T2DM and its associated conditions represent major medical and public health concerns, due to the ever-increasing prevalence of obesity and the subsequent incidence of T2DM and related patient morbidities. Indeed, T2DM is estimated to affect ≅285 million people worldwide, with projections expecting rises to 438 million by 2030 [15]. Consequently, such clinical situations provide significant economic burdens to healthcare providers. In this regard, uncontrolled T2DM is recognised as a mediator of disordered bone metabolism and homeostasis, leading to it becoming a risk factor for the development or exacerbation of osteoporosis, fractures, periodontal disease and orthopaedic/dental implant failure [16–19].

Due to such correlations, numerous in vitro and in vivo model studies have examined the underlying mechanisms by which T2DM influences the metabolic and functional activities of bone cells. Such studies have identified that hyperglycaemic conditions affect all stages of bone repair, with decreased bone formation particularly as a consequence of inhibited osteoblast differentiation and increased apoptosis, induced by oxidative stress and advanced glycation end products (AGEs) [20–25]; coupled with altered ECM deposition, turnover and reduced mineral formation [26]. Consequently, such outcomes culminate in osteopenia and delayed bone healing in vivo [27–29]. Specifically relating to the responses of bone marrow-derived MSCs (BM-MSCs), high glucose environments are established to exhibit reduced proliferative capabilities as a consequence of shortened telomere lengths and early-onset replicative senescence, which impact on MSC properties such as viability, multi-potency, colony-forming efficiency (CFEs) and osteogenic differentiation [30–32]. Impaired osteogenesis under hyperglycaemic conditions occurs at the expense of preferential MSC adipogenic differentiation [21, 22, 33]. However, despite the consensus of studies supporting the deleterious effects of hyperglycaemia on BM-MSC and osteoblastic responses in bone, there are conflicting literature reports suggesting limited effects of high glucose treatment on MSC proliferative capacity and function [34–36]. Such inconsistencies have been confounded by inter-study variations in BM-MSC isolation procedures, the highly variable heterogeneous nature of BM-MSC populations and the glucose concentrations/exposure periods used during in vitro studies. As most in vitro hyperglycaemia studies have utilised BM-MSCs derived from the perivascular niche by bone marrow aspiration and tissue culture plastic adherence [21, 24, 25, 30–32, 35, 36], there is limited information on the influences of hyperglycaemic conditions on the other key MSC populations within the endosteal niche of compact bone (CB-MSCs) [8–10, 12].

Therefore, in order to provide an insight into the influence of high glucose levels on MSCs and associated progenitor cells, heterogeneous CB-MSC populations were compared under normoglycaemic and hyperglycaemic conditions, in terms of their cell proliferative capacity, cellular senescence characteristics, maintenance of stem cell characteristics and differentiation potential towards osteogenic and adipogenic lineages, following extensive culture in vitro. By further understanding the effects and susceptibilities of CB-MSCs from the endosteal niche to hyperglycaemic conditions, we provide additional clarification of the pathophysiology of diabetic bone and importantly, highlight the potential impact of high glucose levels on the metabolism and repair capabilities of CB-MSC populations in situ.

## Results

### High glucose conditions have no negative impact on CB-MSC population doublings and expansion

**Fig. 1:**
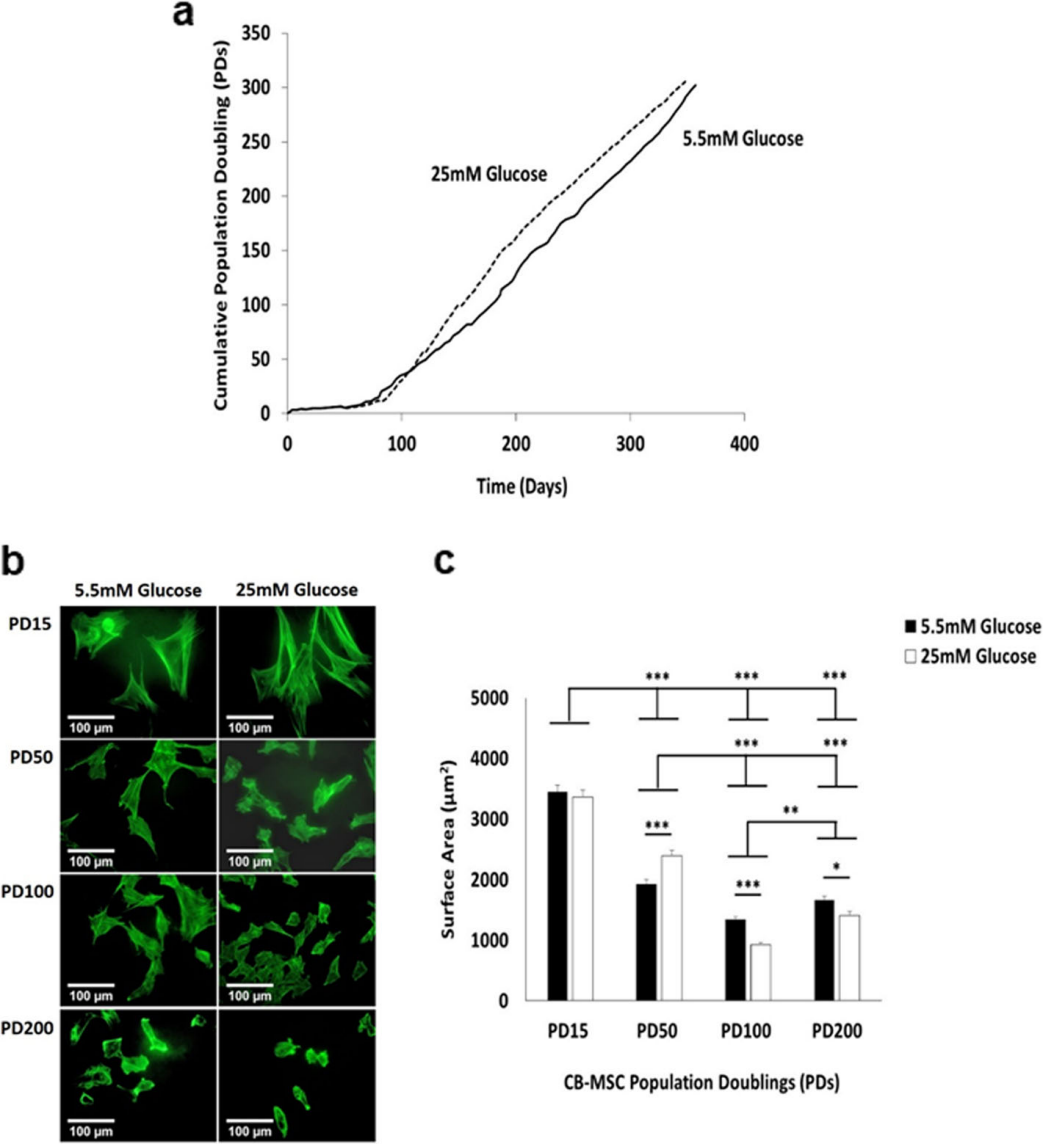
CM-MSC proliferative and morphological characteristics under normoglycaemic and hyperglycaemic conditions. **a** Cumulative population doubling (PDs) of CB-MSCs showed rapid expansion from 10PDs (70–80 days) onwards, with high (25 mM) glucose conditions (*dotted line*), promoting more rapid CB-MSC proliferation rates from 50PDs (~ 120 days) to >300PDs (~ 350 days), versus normal (5.5 mM) glucose levels (*solid line*). **b** CB-MSC morphologies at PD15, PD50, PD100 and PD200 under normoglycaemic and hyperglycaemic conditions, demonstrated morphological changes with culture expansion, but few changes due to high (25 mM) glucose conditions. **c** Quantification of CB-MSC surface areas at PD15, PD50, PD100 and PD200 under normoglycaemic and hyperglycaemic conditions, confirmed limited influences of high (25 mM) glucose conditions on CB-MSC morphologies, versus normal (5.5 mM) glucose levels, except at PD100 and PD200. Significance between CB-MSC populations are shown, ***p < 0.001, **p < 0.01, *p < 0.05. Scale bar = 100 μm

As high glucose conditions have primarily been shown to reduce proliferative capabilities in BM-MSCs due to the induction of replicative senescence [30–32, 37], initial studies assessed the effects of normal (5.5 mM) and high (25 mM) glucose condition on CB-MSC expansion over ~ 350 days in culture, expressed as cumulative PDs (Fig. 1a). CB-MSCs under both normoglycaemic and hyperglycaemic conditions demonstrated a lag phase over the initial 70–80 days in culture (10PDs), followed by rapid exponential growth to >300PDs by ~ 350 days in culture. However, from ~50PDs (~ 120 days in culture) onwards, high glucose conditions were determined to promote more rapid CB-MSC proliferation rates, compared to normal conditions, which persisted for the remaining culture duration without any obvious indications of declining or the onset of cellular senescence.

### Extended CB-MSC culture in high and low glucose conditions alters cell size and morphology

To further examine whether extended CB-MSC expansion over ~ 350 days in culture under normoglycaemic and hyperglycaemic condition induced changes in cell size or morphology, CB-MSC morphological characteristics were assessed by cytoskeletal staining with fluorescein isothiocyanate (FITC)-phalloidin and fluorescence microscopy. CB-MSCs under normal (5.5 mM) and high (25 mM) glucose conditions exhibited considerable alterations in cellular morphologies over the extended culture expansion period (Fig. 1b). CB-MSCs at PD15 under normoglycaemic and hyperglycaemic conditions were shown to particularly possess large stellate-shaped morphologies with the appearance of cytoskeletal stress fibres; with fewer cells with spindle-shaped, fibroblastic-like morphologies. CB-MSCs at PD15 in normal and high glucose conditions were determined to possess the largest surface areas, which were significantly higher than those CB-MSCs at other PDs analysed, irrespective of normal or high glucose treatments (*p* < 0.001, Fig. 1c). At PD50, CB-MSCs morphologies were generally smaller, consisting of cells with more spindle-shaped, fibroblastic or rounded appearances, compared to PD15 (p < 0.001, Fig. 1b-c); although CB-MSCs under hyperglycaemic conditions at PD50 were confirmed to be significantly larger than under normoglycaemic conditions (*p* < 0.001). Similar trends of declining CB-MSC surface areas were evident at PD100 and PD200, with the predominant presence of small stellate and rounded cells with short processes (Fig. 1b-c). CB-MSCs at PD100 had the lowest surface areas of all the populations analysed, particularly under high glucose conditions (p < 0.001). Similar observations were apparent at PD200, with CB-MSCs in hyperglycaemic conditions retaining significantly decreased surfaces areas, compared to CB-MSCs under normoglycaemic conditions (*p* < 0.05).

### Expansion under high glucose conditions have limited impact on the induction of cellular senescence-related markers in CB-MSCs

A number of studies have reported the increased detection of replicative senescence markers in BM-MSCs following culture in high glucose conditions, such as enhanced telomere shortening, positive SA-β-galactosidase staining and elevated expression of tumour suppressor (p53) and cyclin-dependent kinase inhibitor (p21^waf1^, p16^INK4a^) senescence marker genes [30–32, 37]. Consequently, we next asked whether the levels of these and other relevant telomere maintenance markers, rat telomerase (rTERT) and telomerase RNA (TR) [38], changed in CB-MSCs, following expansion over ~ 350 days in culture under normoglycaemic and hyperglycaemic conditions.

**Fig. 2:**
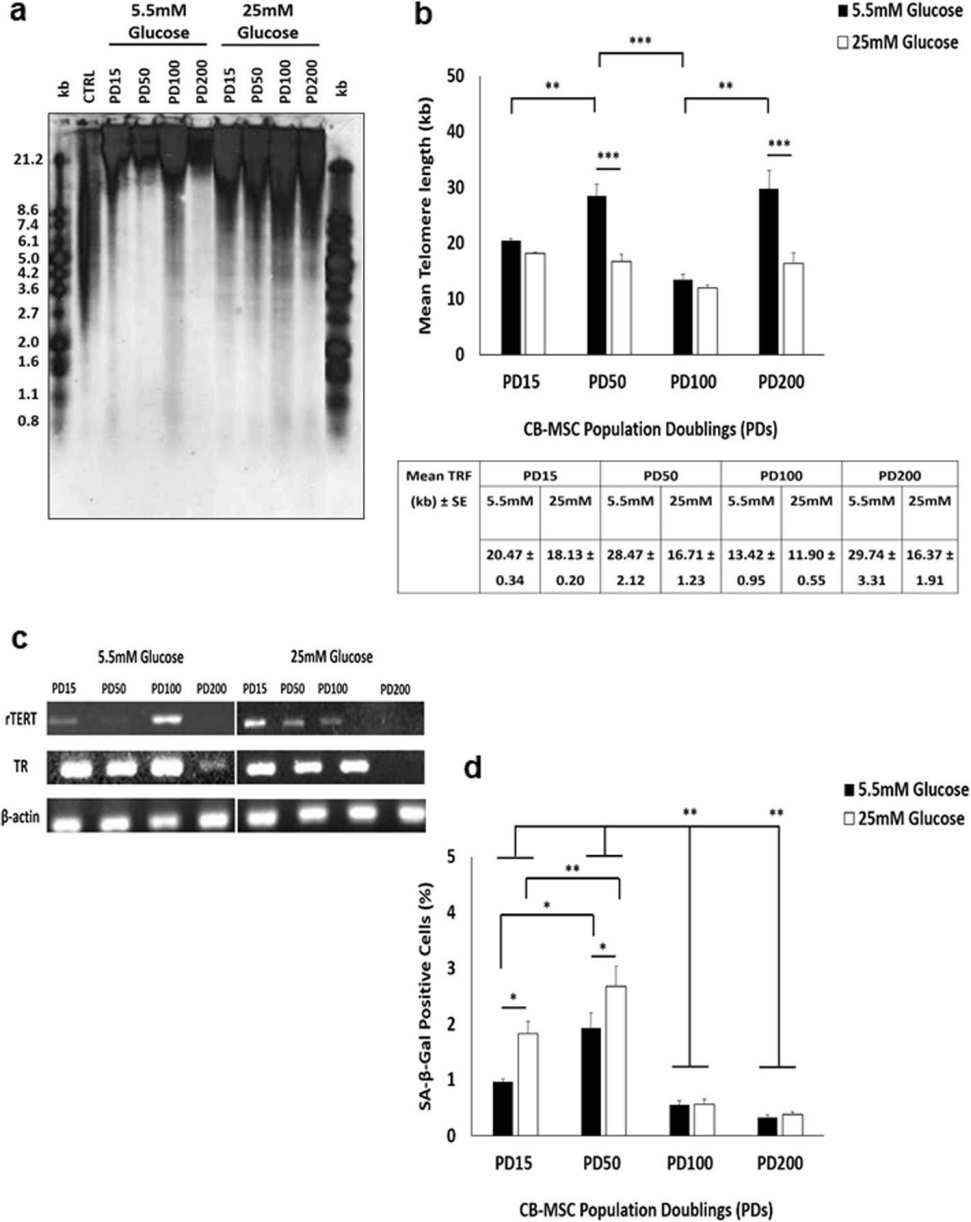
Characterisation of CB-MSC senescence markers under normoglycaemic and hyperglycaemic conditions. **a** Telomere length analysis for CB-MSCs at PD15, PD50, PD100 and PD200, determined by Southern blotting. Right and left hand lanes represent separated DIG-labelled telomere length standards (kb, *in Kit*). CTRL represent telomere length positive control (*in Kit*). **b** Mean telomere lengths calculated for CB-MSCs at PD15, PD50, PD100 and PD200, with values ± SE indicated. **c** Telomere maintenance marker, rTERT and TR, expression at PD15, PD50, PD100 and PD200 under normoglycaemic and hyperglycaemic conditions, by RT-PCR. **d** Percentage SA-β-galactosidase positive CB-MSCs at PD15, PD50, PD100 and PD200 under normoglycaemic and hyperglycaemic conditions, demonstrating low (< 3%) positive staining in CB-MSC populations overall. Significance between CB-MSC populations are shown, ***p < 0.001, **p < 0.01, *p < 0.05

During extended culture in normal (5.5 mM) and high (25 mM) glucose conditions, CB-MSCs demonstrated limited changes in the induction of cellular senescence, although these varied depending on the culture expansion periods assessed. Analyses indicated comparable mean telomere lengths between CB-MSCs at PD15 under normoglycaemic (20.4 kb) and hyperglycaemic (18.1 kb) conditions (*p* > 0.05, Fig. 2a, b). However, whereas mean telomere lengths were significantly longer in CB-MSCs at PD50 in normal glucose conditions (28.4 kb, *p* < 0.01), compared to those at PD15, telomere lengths remained relatively unchanged between PD15 and PD50 in high glucose conditions (16.7 kb, *p* > 0.05). As such, mean telomere lengths were significantly longer at PD50 under normoglycaemic conditions (*p* < 0.001). Significant reductions in telomere lengths were identified at PD100 under normoglycaemic conditions (14.4 kb), compared to PD50 (p < 0.001); although again, non-significant reductions in telomere length were detected between PD50 and PD100 (11.9 kb), under hyperglycaemic (*p* > 0.05). As evident at PD50, mean telomere lengths were significantly longer in CB-MSCs at PD200 in normal glucose conditions (29.7 kb, *p* < 0.01), compared to those at PD100. However, telomere lengths remained relatively unchanged in CB-MSCs at PD200 in high glucose conditions (16.3 kb, p > 0.05), compared to PD100. Consequently, telomere lengths were significantly longer at PD200 under normoglycaemic conditions (*p* < 0.001).

Intriguingly, mean telomere lengths showed few correlations with the detection of telomere maintenance markers, rTERT and TR (Fig. 2c). rTERT was particularly expressed in CB-MSCs under normoglycaemic conditions at PD15 and PD100, with trace rTERT levels at PD50. In contrast, rTERT expression gradually declined between PD15 and PD100 in CB-MSCs under hyperglycaemic conditions. rTERT was undetectable in CB-MSCs at PD200, in both normal and high glucose conditions. TR expression was similar in CB-MSCs at PD15, PD50 and PD100 under both normoglycaemic and hyperglycaemic conditions (Fig. 2c). However, TR detection declined in CB-MSCs at PD200.

Further analyses indicated that despite significant differences in telomere lengths between CB-MSCs in normal and high glucose conditions at certain stages during culture expansion, SA-β-galactosidase staining confirmed that the majority of cell present in normoglycaemic and hyperglycaemic conditions were non-senescent, indicated by < 3% of CM-MSCs per 30 μm^2^ staining positive (Fig. 2d). This was supported by their retained abilities to achieve PDs well above the established PD limit for senescent induction at < 0.5PD/week [12, 39] (Fig. 1a). However, despite such low numbers overall, higher proportions of senescent cells were present in CB-MSCs at PD15 and PD50 under hyperglycaemic conditions (*p* < 0.05 at PD15), compared to normoglycaemic conditions (Fig. 2d). Furthermore, significantly higher senescent cell numbers were identified at PD50 under both conditions, versus CB-MSCs at PD15 in normal glucose concentrations (p < 0.05 and *p* < 0.01, respectively). CB-MSCs at both PD100 and PD200 exhibited the lowest proportions of senescent cells, being significantly lower than CB-MSCs at PD15 and PD50, under both conditions (p < 0.01).

**Fig. 3:**
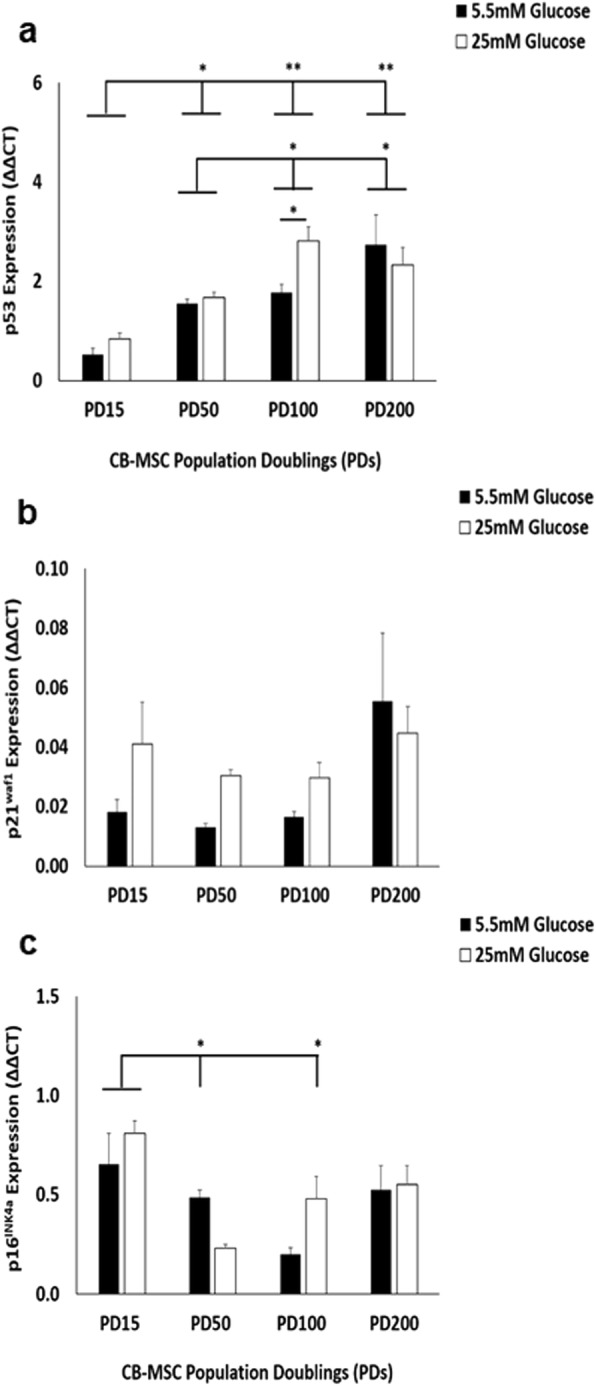
Characterisation of CB-MSC senescence marker (p53, p21^waf1^ and p16^INK4a^) expression under normoglycaemic and hyperglycaemic conditions. **a** Significant differences in p53 gene expression were shown between PD15 and PD50, PD100 and PD200 under normoglycaemic and hyperglycaemic conditions. However, only CB-MSCs at PD100 demonstrated significant higher p53 expression in high glucose, compared to normal glucose conditions. **b** p21^waf1^ exhibited very low expression, with no significant differences in expression between normoglycaemic and hyperglycaemic conditions. **c** p16^INK4A^ expression was low across all PDs, although CB-MSCs at PD15 under both normoglycaemic and hyperglycaemic conditions demonstrated highest p16^INK4A^ expression, which declined by PD50 onwards. Significance between CB-MSC populations are shown, **p < 0.01, *p < 0.05

Analysis of cellular senescence-related genes (p53, p21^waf1^ and p16^INK4a^, Fig. 3a-c respectively), showed that all CB-MSCs expressed the tumour suppressor gene, p53 and downstream gene, p21^waf1^ (regulator of cell cycle progression at G1 and S phase). p53 demonstrated a steady increase in gene expression from PD15 until PD100, which remained expressed at high levels at 200PD. Consequently, significant differences in p53 gene expression were shown between PD15 and PD50, PD100 and PD200 under normoglycaemic and hyperglycaemic conditions (*p* < 0.05, Fig. 3a). However, only CB-MSCs at PD100 demonstrated significant higher p53 expression in high glucose, compared to normal glucose conditions (p < 0.05). In contrast, p21^waf1^ exhibited very low expression in all experimental groups, with no significant differences in expression shown (*p* > 0.05, Fig. 3b). Expression of another recognised tumour suppressor gene, p16^INK4A^, was also generally low across all PDs, although CB-MSCs at PD15 under both normoglycaemic and hyperglycaemic conditions demonstrated highest p16^INK4A^ expression, which generally declined by PD50 onwards (p < 0.05 versus PD50 under normoglycaemic and PD100 under hyperglycaemic conditions, Fig. 3c).

### Extended CB-MSC culture in high and low glucose conditions has limited impact on stem cell marker expression, but alters colony forming efficiencies

Many studies have indicated that high glucose conditions and/or the induction of cellular senescence promotes the loss of stem cell characteristics in MSC populations, such as stem cell marker expression and CFEs [12, 30–32, 39]. Therefore, we next investigated whether the expression of various mesenchymal, haematopoietic, neural crest and pluripotent stem cell markers changed in CB-MSCs, following expansion over ~ 350 days in culture under normoglycaemic and hyperglycaemic conditions. Similarly, we also assessed whether the CFEs of CB-MSCs were influenced by such conditions.

**Fig. 4:**
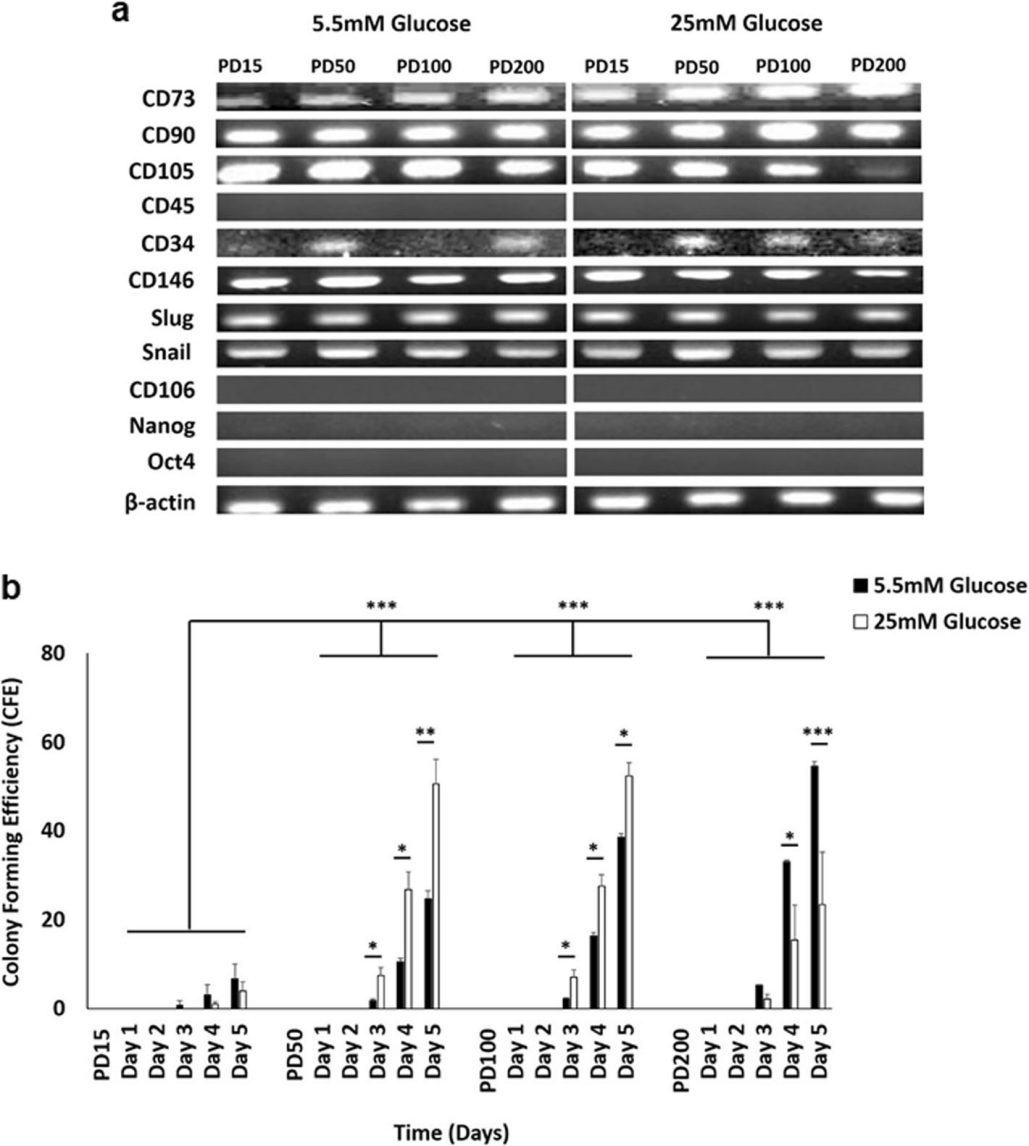
Characterisation of CB-MSC stem cell properties under normoglycaemic and hyperglycaemic conditions. **a** CB-MSCs at PD15, PD50, PD100 and PD200 under normoglycaemic and hyperglycaemic conditions, expressed CD73, CD90 and CD105; and were negative for CD45. CD105 expression appeared lost at PD200 under hyperglycaemic conditions. CB-MSCs also expressed low levels of CD34 throughout culture expansion, irrespective of normoglycaemic/hyperglycaemic conditions. CD146, Slug and Snail were expressed throughout expansion under normoglycaemic/hyperglycaemic conditions. CD106, Nanog and Oct4 expression were undetectable. **b** Colony forming efficiencies (CFEs) for CB-MSCs at PD15 had significantly lower efficiencies, compared to PD50, PD100 and PD200, in both normoglycaemic and hyperglycaemic conditions. CFEs at PD50 and PD100 were significantly higher under hyperglycaemic, compared to normoglycaemic conditions (days 3–5). CFEs were significant decreased at PD200 by hyperglycaemia, compared to normoglycaemic conditions (days 4–5). Significance between CB-MSC populations are shown, ***p < 0.001, **p < 0.01, *p < 0.05

RT-PCR analysis confirmed that CB-MSCs at PD15, PD50, PD100 and PD200 under normoglycaemic and hyperglycaemic conditions, positively expressed the mesenchymal progenitor cells surface antigens, CD73 (ecto-5-nucleotidase), CD90 (Thy-1) and CD105 (endoglin) and were found to be negative for the expression of CD45 (hematopoietic surface antigens) (Fig. 4a). However, CD105 expression appeared to be lost at PD200 under hyperglycaemic conditions. CB-MSCs also expressed low levels of CD34 throughout culture expansion, irrespective of normoglycaemic/hyperglycaemic conditions. Consistent expression of CD146 and embryonic stem cell markers, Slug and Snail; were also evident throughout culture expansion under normoglycaemic/hyperglycaemic conditions. In contrast, expression of CD106 (VCAM-1) and pluripotent stem cell markers, Nanog and Oct4, were undetectable in CB-MSCs.

CFEs indicated that CB-MSCs at PD15 had significantly lower efficiencies, compared to cells at PD50, PD100 and PD200, irrespective of normoglycaemic or hyperglycaemic conditions (all *p* < 0.001, Fig. 4b). In addition to the significant increases in CFEs at PD50 and PD100, these were shown to be significantly higher under hyperglycaemic conditions on days 3–5 (*p* < 0.01–0.05), compared to normoglycaemic conditions. However, significant decreases in CFEs were identified at PD200 under hyperglycaemic conditions on days 4–5 (p < 0.001–0.05), compared to normoglycaemic conditions.

### High glucose conditions significantly inhibit CB-MSC osteogenic differentiation, as does extended culture expansion under high and low glucose conditions

As high glucose conditions have been shown to negatively impact on the osteogenic differentiation capabilities of BM-MSCs [21, 22, 30–33], we next compared the effects of normoglycaemic and hyperglycaemic conditions on CB-MSC osteogenic capabilities over ~ 350 days in culture.

**Fig. 5:**
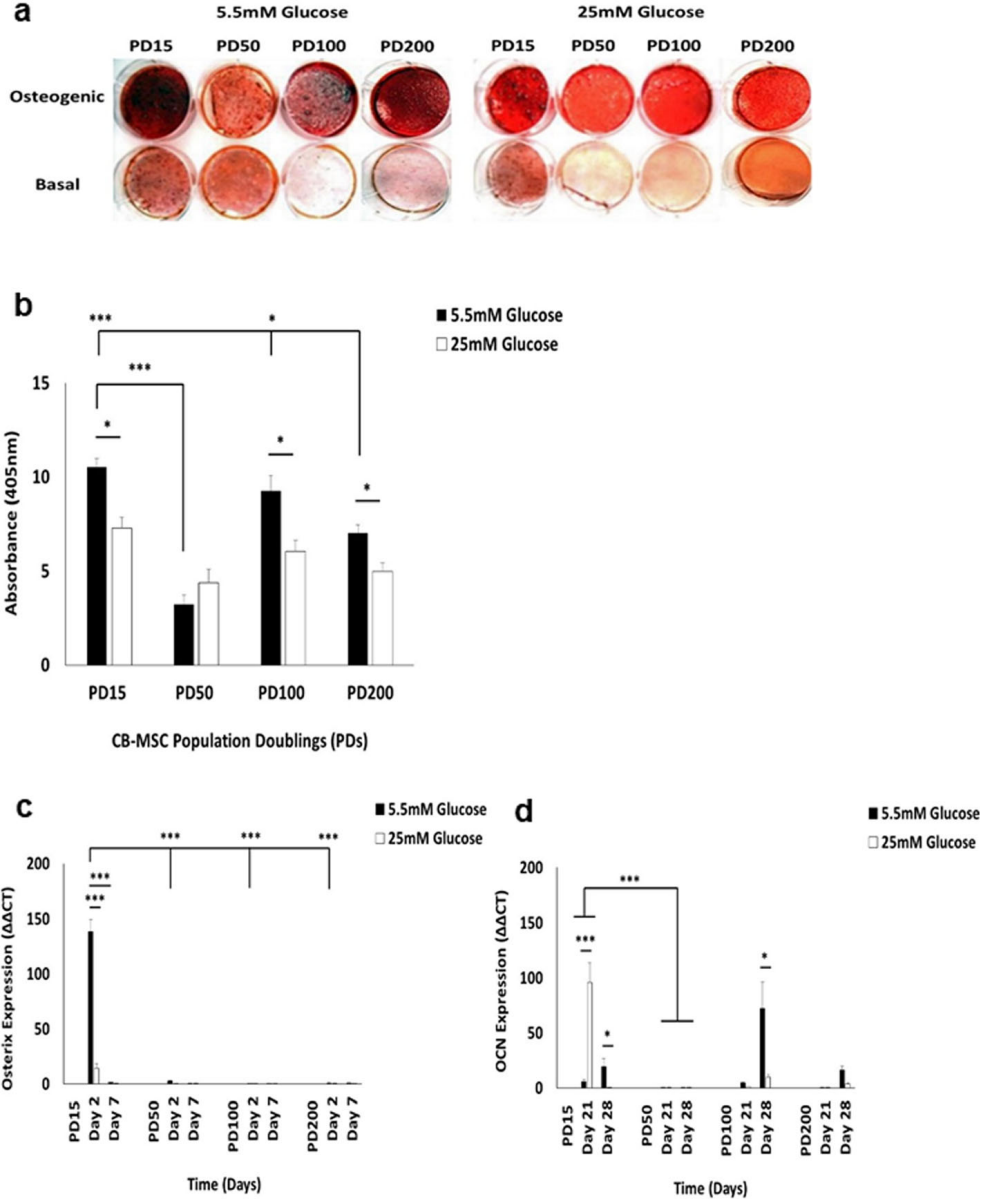
Characterisation of CB-MSC osteogenic differentiation under normoglycaemic and hyperglycaemic conditions. **a** Mineral deposition, as visualised by Alizarin Red staining, by CB-MSCs cultured for 28 days in osteoinductive and basal (control) medium. Hyperglycaemia inhibited CB-MSC osteogenic differentiation at PD15, PD100 and PD200, but not at PD50. **b** Quantification of Alizarin red staining by CB-MSCs cultured for 28 days in osteoinductive medium, confirming significantly reduced staining at PD15, PD100 and PD200 under hyperglycaemic conditions, but not at PD50. Assessment of **c** early (osterix) and **d** late (OCN) osteogenic marker expression by CB-MSCs under normoglycaemic and hyperglycaemic conditions, by Q-PCR. CB-MSCs at PD15 under normoglycaemic conditions exhibited significant increased osterix expression at day 2, compared to PD50, PD100 and PD200. Hyperglycaemic conditions significantly reduced osterix expression at day 2. CB-MSCs at PD15 exhibited significantly elevated OCN expression under hyperglycaemic conditions at day 21. Although OCN expression declined by day 28, significantly elevated OCN expression was shown at PD15 under normoglycaemic conditions. OCN expression was severely impaired at PD50 under both normal and high glucose conditions. Hyperglycaemic conditions exerted no significant effects on OCN expression at PD100 and PD200, although significantly increased expression was evident at PD100 under normal glucose conditions, at day 28. Significance between CB-MSC populations are shown, ***p < 0.001, *p < 0.05

CB-MSCs cultured in osteogenic medium under normal (5.5 mM) and high (25 mM) glucose conditions for 28 days, demonstrated distinct formation of mineralised bone nodules that stained positive with Alizarin red (Fig. 5a). However, CB-MSCs in non-osteogenic basal (control) media demonstrated minimal or no staining. At PD15, CB-MSCs demonstrated distinct Alizarin red positive staining in osteogenic medium under normoglycaemic conditions, but marked decreases in Alizarin red staining were observed in CB-MSCs at PD50, indicating an overall impairment in CB-MSC osteogenic capabilities. Although Alizarin red staining positivity was partly restored in normal glucose conditions at PD100, further reductions in Alizarin red staining were shown at PD200. When compared to CB-MSCs cultured in osteogenic medium under hyperglycaemic conditions, consistently less Alizarin red staining was evident at all PDs examined, versus normoglycaemic conditions. Alizarin red staining differences were confirmed spectrophotometrically (Fig. 5b), with CB-MSCs at PD15 under normal glucose conditions exhibiting significantly higher stain quantification than at PD50 (*p* < 0.001). Despite no significant differences in Alizarin red stain quantification being identified at PD100, compared to CB-MSCs at PD15 (*p* > 0.05), CB-MSCs at PD200 demonstrated significantly reduced mineral deposition under normoglycaemic conditions, versus PD15 and PD100 (*p* < 0.001 and *p* < 0.05, respectively). In line with the Alizarin red histology data, hyperglycaemic conditions induced significantly reduced Alizarin red stain quantification compared to normoglycaemic controls, at PD15, PD100 and PD200 (all p < 0.05), although no significant decreases were evident at PD50 (*p* > 0.05).

Next, we confirmed these results by analysis of osteogenic marker (osterix and osteocalcin, OCN) expression by Q-PCR. Osterix is regarded as an early expressed gene during osteogenic differentiation and thus, was quantified at day 2 and day 7. CB-MSCs at PD15 under normoglycaemic conditions exhibited significant increased osterix expression at day 2, compared to PD50, PD100 and PD200 (all *p* < 0.001, Fig. 5c). Conversely, hyperglycaemic conditions induced significant reductions in osterix expression at day 2, versus normal glucose conditions (p < 0.001). Although osterix expression was downregulated by CB-MSCs at PD15 in normoglycaemic conditions at day 7 (p < 0.001), no significant differences in osterix expression between day 2 and day 7 were evident at PD50, PD100 or PD200 (all *p* > 0.05).

Expression of the late 'osteogenic marker, OCN, was quantified at day 21 and day 28. At PD15, CB-MSCs exhibited significantly elevated OCN expression under hyperglycaemic conditions at day 21 (p < 0.001, Fig. 5d). Although OCN expression declined in high glucose conditions by day 28, significantly elevated OCN expression was shown for CB-MSCs at PD15 under normoglycaemic conditions (*p* < 0.05). In contrast, OCN expression was severely impaired at PD50 under both normal and high glucose conditions, compared to PD15 (p < 0.001); with no significant differences in expression induced by hyperglycaemia (*p* > 0.05). Whilst OCN expression for CB-MSCs at PD100 and PD200 were similar to that induced at PD15 at day 21, hyperglycaemic conditions exerted no significant differences on expression, compared to normoglycaemic conditions. However, significant increases in OCN expression by CB-MSCs at PD100 were evident under normal glucose, compared to high glucose conditions, at day 28 (p < 0.05), although no significant expression differences were identified at PD200 (p > 0.05).

### High glucose conditions significantly inhibit CB-MSC adipogenic differentiation, as does extended culture expansion under high and low glucose conditions

As impaired BM-MSC osteogenic differentiation under high glucose conditions has been associated with preferential adipogenic differentiation [21, 22, 33], we next determined whether similar inductions in CB-MSC adipogenic capabilities were induced under normoglycaemic and hyperglycaemic conditions over ~ 350 days in culture.

**Fig. 6:**
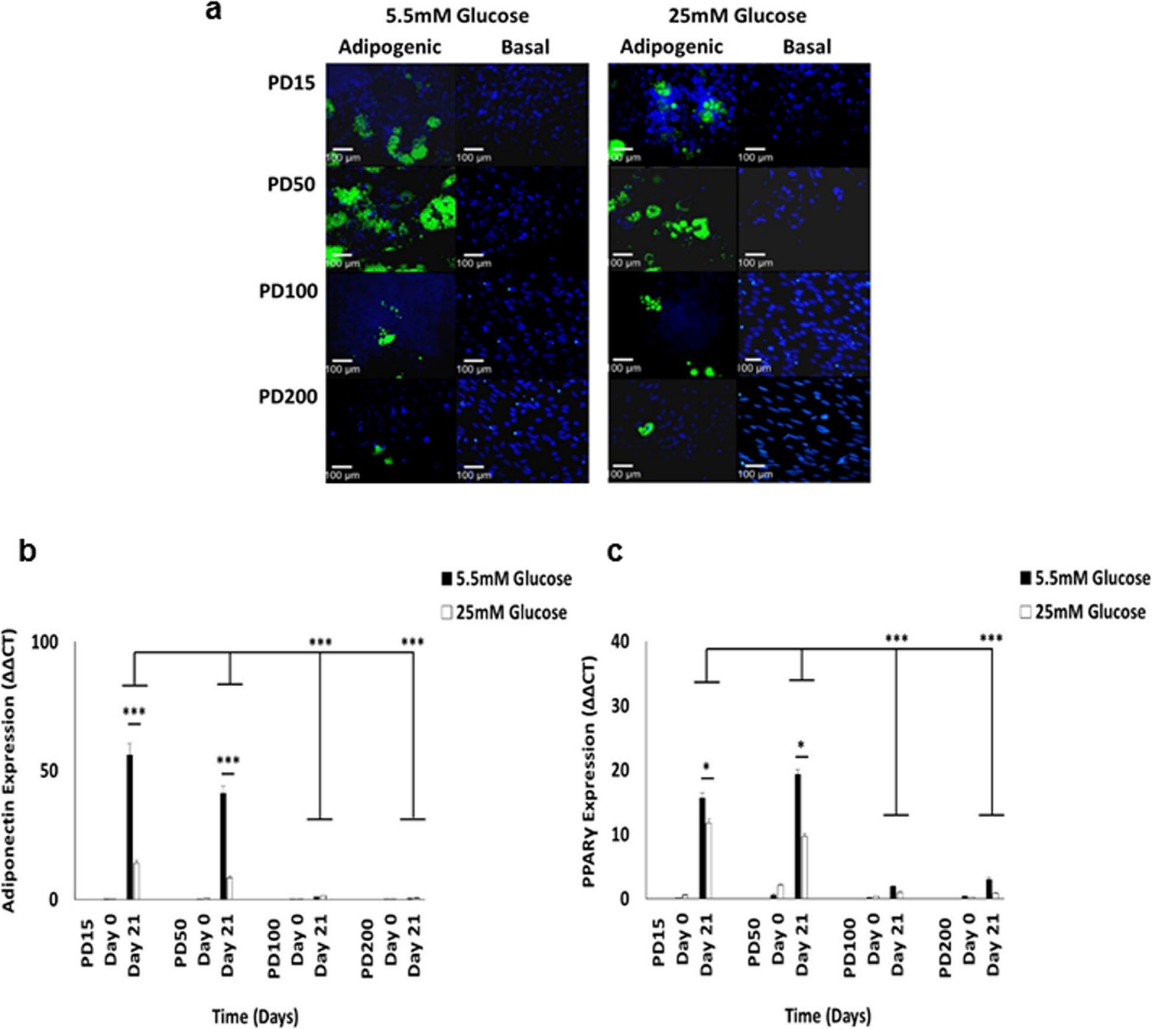
Characterisation of CB-MSC adipogenic differentiation under normoglycaemic and hyperglycaemic conditions. **a** Lipid vacuole formation, as visualised by LipidTOX™ Green Neutral Lipid Stain, by CB-MSCs cultured for 21 days in adipogenic and basal (control) medium. At PD15 and PD50, CB-MSCs demonstrated prominent LipidTOX™ staining under both normoglycaemic and hyperglycaemic conditions. CB-MSCs at PD100 and PD200 exhibited fewer lipid vacuoles and a reduced capacity to differentiate into adipocytes. Assessment of late adipogenic marker, **b** adiponectin and **c** PPARγ, expression by CB-MSCs under normoglycaemic and hyperglycaemic conditions, by Q-PCR. Although baseline (day 0) analyses demonstrated no differences in adiponectin or PPARγ expression between normal and high glucose conditions, both showed significantly increased expression by CB-MSCs at PD15 and PD50 under normoglycaemic conditions at day 21. Significantly reduced adiponectin and PPARγ expression were identified at PD100 and PD200, under both normal and high glucose conditions. Significance between CB-MSC populations are shown, ***p < 0.001, *p < 0.05. Scale bar = 100 μm

CB-MSCs cultured in adipogenic medium under normal (5.5 mM) and high (25 mM) glucose conditions for 21 days, demonstrated the formation of large lipid droplets that stained positive with LipidTOX™ Green Neutral Lipid Stain (Fig. 6a). In contrast, CB-MSCs in non-adipogenic basal (control) media demonstrated minimal or no staining. At PD15 and PD50, CB-MSCs demonstrated prominent clusters of positive LipidTOX™ staining under both normoglycaemic and hyperglycaemic conditions, whereas CB-MSCs at PD100 and PD200 exhibited fewer lipid vacuoles and a reduced capacity to differentiate into adipocytes.

Next, we verified these findings results by analysis of late adipogenic marker (adiponectin and peroxisomal proliferator-activated receptor-γ, PPARγ) expression by Q-PCR (Fig. 6b-c, respectively). Although baseline (day 0) analyses demonstrated no significant differences in adiponectin or PPARγ expression between normal and high glucose conditions (all *p* > 0.05), both markers showed significantly upregulated expression by CB-MSCs at PD15 and PD50 under normoglycaemic conditions at day 21 (*p* < 0.001 and *p* < 0.05, respectively). Conversely, significant reductions in adiponectin and PPARγ expression were noted in CB-MSCs at PD100 and PD200, under both normal and high glucose conditions (p < 0.001), resulting in no significant differences in adiponectin or PPARγ expression between normoglycaemic and hyperglycaemic conditions at these PDs (all *p* > 0.05).

## Discussion

This in vitro study examined the long-term effects of hyperglycaemic culture conditions on the properties and behaviour of heterogeneous CB-MSC populations, derived from the endosteal niche of compact bone [8–10]. The study provides novel information supplementing previous investigations into MSC populations from the bone marrow stroma. By focussing on CB-MSCs, we have evaluated the biological effects of hyperglycaemia on a more mature, committed MSC population, with prominent roles in bone repair and remodelling [9, 12].

Our data suggests that prolonged expansion under persistent hyperglycaemic conditions had limited impact on the proliferative capabilities of heterogeneous CB-MSC populations as a whole, actually stimulating proliferation between 50-300PDs. Consequently, such findings are counterintuitive to the consensus of hyperglycaemic conditions having negative influences on MSC and osteoblast behaviour [20–26], including increased susceptibility to telomere-dependent, replicative senescence [30–33]. Nonetheless, in line with high glucose conditions promoting CB-MSC PDs, similar conclusions of hyperglycaemia having positive or negligible effects on BM-MSC proliferation rates have also been reported [34–36].

The lack of negative hyperglycaemic effects on CB-MSC proliferation were confirmed by cells retaining similar morphologies throughout culture expansion in normoglycaemic and hyperglycaemic conditions, the low numbers of SA-β-galactosidase positive cells and the limited expression of senescence markers, p53, p21^waf1^ and p16^INK4a^. As evident here, similar decreases in cell size, morphologies and cytoskeletal characteristics have previously been reported with heterogeneous rat BM-MSC populations following extensive in vitro culture expansion [40]. Although CB-MSCs exhibited increased p53 expression with long-term expansion, only at PD100 were significant increases in p53 expression evident under hyperglycaemic conditions. MSC replicative senescence is acknowledged as a multi-step process driven by p53, which promotes growth arrest by inducing p21^waf1^ expression, inhibiting G_1_-S phase progression [37]. p53 and p21^waf1^ act as tumour suppressors to regulate cell proliferation at rates that provide genomic stability during MSC division [41]. p53 is also a negative regulator of pre-osteoblast and pre-adipocyte formation. Therefore, p53 and p21^waf1^ expression regulate MSC expansion in an undifferentiated state. MSC telomere erosion can also initiate p16^INK4a^ checkpoints, suppressing proliferation and inducing senescence [42]. Consequently, both p53 and p16^INK4a^ are regarded as the principal mediators of MSC senescence [43, 44]. However, despite elevated p53 expression in long-term CB-MSC culture, the low p21^waf1^ and p16^INK4A^ expression and lack of induction by hyperglycaemia, would imply the absence of any significant promotion of premature CB-MSC senescence under such conditions.

Maintenance of CB-MSC telomere lengths would be facilitated by the detection of rTERT and TR during extended culture expansion [12, 38], which remained relatively consistent under normoglycaemic and hyperglycaemic conditions until PD200. Although marker levels generally discounted the overall presence of enhanced senescent CB-MSC populations as a whole with increased glucose exposure, telomere lengths exhibited limited correlations with other senescence markers. Indeed, telomere length profiles varied considerably during extended culture expansion under both normoglycaemic and hyperglycaemic conditions, with CB-MSCs exposed to high glucose conditions being unable to enhance mean telomere lengths at PD50 and PD200, leading to significantly decreased telomeres overall. However, significant telomere length reductions were also identified at PD100 under normoglycaemic conditions. Such findings are likely to be associated with the heterogeneous nature of CB-MSCs, with the loss of minor, more senescent CB-MSC sub-population(s) with inferior telomere dynamic characteristics at various stages during prolonged expansion [12, 39]. Our previous work has demonstrated that CB-MSCs at PD15 contained immature/undifferentiated MSCs, in addition to osteoprogenitor cell and post-mitotic, bone lining osteoblast sub-population(s). However, these mature osteoblasts were lost by PD50 leading to a more predominant immature MSC population overall [12]. Therefore, such changes in sub-population composition may reflect the reductions in telomere length profiles obtained during extended culture expansion, especially under hyperglycaemic conditions. As rTERT inactivation by hyperglycaemia is unlikely to contribute to the inability of CB-MSCs at PD50 and PD200 to maintain telomere lengths at equivalent levels to normoglycaemic conditions [45, 46], it appears that particular CB-MSC sub-population(s) are more susceptible to telomere shortening and replicative senescence in high glucose conditions, in line with reports with BM-MSCs [30–32]. However, these sub-population(s) provide relatively insignificant contributions to the proliferative capabilities of heterogeneous CB-MSC populations overall, which are lost during extended culture expansion.

Although it has been demonstrated that extensive MSC expansion in vitro may lead to the loss of stem cell properties and malignant transformation [47, 48], the proliferative and senescence findings indicated that heterogeneous CB-MSC populations under both normoglycaemic and hyperglycaemic conditions remain non-transformed during culture expansion. While the proliferative capacities of most cell types are finite, previous studies involving heterogeneous MSC populations derived from rodent tissues have reported that such cultures retain untransformed states, despite achieving > 100 passages without evidence of cell cycle arrest [40, 49]. Such conclusions are based on the increased expression of p53 with long-term expansion [37, 41, 50]. Constitutive expression of rTERT and TR throughout culture expansion also implies that heterogeneous CB-MSC populations are transformed [51]. However, unlike humans, rodent cells retain endogenous TERT competency throughout their lifespans and consequently do not exhibit classical replicative senescence, leading to longer telomeres overall [52]. Indeed, positive TERT expression in human MSCs is associated with the retention of stem cell properties and inhibition of spontaneous differentiation [53].

Further studies examined the ability of CB-MSCs in normal and high glucose conditions to maintain stem cell marker expression, form colonies and undergo osteogenic and adipogenic differentiation. In line with previous findings, CB-MSCs were positive for MSC markers, CD73, CD90 and CD105; and negative for haematopoietic stem cell marker, CD45; although positive CD34 expression has been previously reported in CB-MSCs [12]. Neural crest markers, Slug and Snail, were also expressed at all PDs under normoglycaemic and hyperglycaemic conditions, as was CD146. Slug and Snail are implicated in promoting commitment to a mesenchymal lineage [54, 55], whilst CD146 is an established marker of MSC proliferation and multi-potency [56]. Therefore, expression of these markers may be associated with continued CB-MSC proliferation during extended culture, suggesting a level of immaturity and multi-potency capabilities in some sub-populations. Consequently, in corroboration of CB-MSCs being resistant to hyperglycaemia inducing premature replicative senescence, marker expression and stem cell characteristics remained relatively stable throughout the extended culture expansion period; whereas stem cell marker expression tends to decline in truly senescent MSC populations [39, 57].

CB-MSCs at PD15 had significantly lower CFEs, compared to those displayed by CB-MSCs at PD50, PD100 and PD200; irrespective of normoglycaemic or hyperglycaemic conditions. Such phenomena are suggested to be related to CB-MSCs at PD15 containing sub-population(s) of low proliferative, post-mitotic/committed pre-osteoblasts and osteoblasts from the bone lining cells of the endosteum [12]. CFEs at PD50 and PD100 were also significantly higher under hyperglycaemic conditions, although hyperglycaemia reduced CFEs at PD200. Therefore, at PD50 and PD100, hyperglycaemic conditions were stimulatory to CFEs, rather than inhibitory in line with the induction of replicative senescence [30–32]; whereas it is only at 200PDs where high glucose conditions appear to be exerting any negative effects on CFEs.

In this study, hyperglycaemia was shown to exert inhibitory effects on the osteogenic differentiation of heterogeneous CB-MSC populations at PD15, PD100 and PD200, evident by decreased mineral deposition and altered osteogenic marker expression in high glucose conditions. However, this was not the case at PD50, where in line with previous findings, marked decreases in mineral staining and osteogenic marker expression were evident with CB-MSCs at PD50 under normoglycaemic and hyperglycaemic conditions [12]. The particularly high osteogenic capacity of CB-MSCs at PD15 under normoglycaemic conditions are a likely consequence of the presence of more mature/committed osteoprogenitor and pre-osteoblastic stromal cells, proposed to be present within the endosteal niche [9, 10, 12]. Impaired osteogenic responses at PD50 suggest a change in the overall composition of the heterogeneous CB-MSC population between PD15 and PD50, to one predominantly consisting of more immature, multi-potent MSC populations [12]. As such, the delayed induction of osterix expression, coupled with the early onset of OCN expression, suggests perturbations to normal ECM formation and mineralisation within high glucose environments, especially at PD15 [23, 58, 59]. In contrast, fewer differences in mineral content and osterix/OCN expression were evident between normoglycaemic and hyperglycaemic conditions at PD50, PD100 or PD200, possibly due to extended culture conditions impeding normal CB-MSC osteogenic capabilities at higher PDs influencing ECM deposition, even under normal glucose conditions. Similar findings of high glucose conditions inhibiting osteogenic differentiation, bone ECM deposition and overall mineralisation have been reported previously, with lesser quality bone formed [20–32].

In terms of adipogenesis, CB-MSCs at PD15 and PD50 demonstrated similar LipidTOX™ staining indicative of adipogenic capabilities irrespective of normoglycaemic or hyperglycaemic conditions, although expression of both adiponectin and PPARγ were significantly impaired in high glucose conditions. Such findings are partly counterintuitive to previous reports that CB-MSCs demonstrate enhanced adipogenic induction at PD50, compared to PD15 [12]. Nonetheless, the increased osteogenic commitment of CB-MSC at PD15 did not negate adipogenic differentiation, implying the additional presence of immature cell sub-population(s) with adipogenic capabilities. Despite abrogated osteogenic responses at PD50, CB-MSCs exhibited improved adipogenic differentiation, possibly due to the absence of committed osteoprogenitor cells, enabling immature cell differentiation into adipocytes. In contrast, CB-MSCs at PD100 and PD200 exhibited reduced adipogenesis overall. Consequently, no differences in adipogenic marker expression were evident between CB-MSCs in normal and high glucose conditions at PD100 and PD200, indicating the disruption of CB-MSC adipogenic capabilities at higher PDs. It is established that hyperglycaemia can alter gene expression and drive MSC differentiation towards the adipogenic lineage [21, 22, 33]. Therefore, CB-MSC adipogenic responses at all PDs analysed are mostly contradictory to this phenomenon, given the comparable or impaired LipidTOX™ staining and adipogenic marker expression identified herein under hyperglycaemic conditions; although failed adipogenic differentiation as evident at PD100 and PD200 has previously been reported following extended in vitro culture expansion [60]. As increases in adipogenic capabilities are often associated with impaired osteogenesis, the collective conclusions on the abilities of hyperglycaemia to influence CB-MSC osteogenic/adipogenic differentiation are that high glucose conditions can inhibit osteogenic differentiation, although this does not necessarily increase adipogenesis.

The reasons behind increased CB-MSCs susceptibilities to disrupted osteogenic (at PD50 and PD200) and adipogenic (at PD100 and PD200) differentiation, remain to be elucidated. However, factors such as additional alterations to osteogenic/adipogenic gene expression other than those evaluated here, CB-MSC populations possessing lower mean telomere lengths (such as PD100); or CB-MSCs at PD50 being less lineage restricted due to the loss of more mature/committed sub-population(s) present at earlier PDs, are all plausible explanations for the dysfunctional osteogenesis/adipogenesis observed. T2DM is commonly associated with insulin resistance, hyperinsulinemia and metabolic disturbances leading to altered cell signalling and genotypic/phenotypic responses [13, 14]. Although insulin stimulates osteogenic proliferation, differentiation and bone formation via insulin growth factor (IGF) receptor binding and downstream signalling pathway activation [61], such responses differ in T2DM due to insulin resistance and absence of IGF receptor stimulation. Indeed, T2DM patients treated with insulin have an increased risk of fracture development, related to compromised bone strength [62]. Consequently, the impact of hyperglycaemic conditions on the levels and signalling mechanisms of insulin and other key bone repair-related mediators in CB-MSCs, such as IGFs, transforming growth factor-β_1_ (TGF-β_1_) and bone morphogenic proteins (BMPs), warrant further investigation; especially in terms of whether disruptions to such signalling pathways contribute to impaired osteogenic/adipogenic differentiation capabilities in CB-MSCs.

It has further been proposed that high glucose effects on MSC populations differ depending on their levels of stemness and whether TA cells or more lineage restricted MSC populations are present [34]. Asymmetric cell division by MSCs produces highly proliferative, immature TA cells, capable of forming colonies and multi-potency; alongside more quiescent, mature TA cell populations with lower colony forming efficiencies, more restricted lineage potentials [11, 12, 39]. As such, despite proliferative responses being largely unaffected by extended culture expansion, CB-MSCs at PD15 represent a pool of more committed and lineage restricted cell populations, which are subsequently lost during expansion at PD50, PD100 and PD200 under normoglycaemic and hyperglycaemic conditions, leading to a predominance of more multi-potent immature TA cells with delayed or impaired osteogenic and adipogenic differentiation capabilities [9, 10, 12]. Consequently, as lineage restricted MSCs are proposed to be the first responders during mineralised tissue repair, prolonged expansion may influence the overall compositions of CB-MSC populations with key roles in mediating bone healing.

## Conclusions

Although MSCs have key roles in bone repair, hyperglycaemia associated with T2DM can impair healing. This study has demonstrated that unlike previous reports on the increased susceptibility of other BM-MSCs to hyperglycaemic conditions, heterogeneous CB-MSC populations derived from the endosteal niche of compact bone are largely unaffected by prolonged exposure to high glucose conditions, in terms of their proliferative responses and the retention of stem cell characteristics. However, osteogenic and adipogenic differentiation responses were significantly impaired under high glucose conditions. Despite the heterogeneous CB-MSC populations being collectively resistant to detrimental hyperglycaemic effects, minor more senescent CB-MSC sub-population(s) showed increased susceptibility to hyperglycaemia. Although the loss of these sub-population(s) during extended culture expansion exerted relatively little impact on the proliferative or stem cell characteristics of CB-MSCs overall, their absence negatively impacted on osteogenic and adipogenic differentiation capabilities. Whereas previous reports have mostly focused on BM-MSCs, this study provides a unique insight into the influence of high glucose levels on heterogeneous CB-MSC populations. The data presented provides an important initial characterisation of these effects, leading to a better understanding of the potential impact of high glucose levels on the metabolism and repair capabilities of CB-MSC populations in situ and the pathophysiology of diabetic bone; which has significant impacts on the clinical management of orthopaedic and dental conditions, due to the ever-increasing incidence of T2DM worldwide.

## Methods

### Isolation of CB-MSCs and culture expansion under high and low glucose conditions

CB-MSCs were isolated from 28-day old male Wistar rat femur and humerus bones, as previously described [12]. Wistar rats (110–130 g*, n = 4*) were obtained from the colony maintained by Charles River (Margate, UK). Animals were housed in individual cages under standard light/dark cycle, with access to food (standard chow) and water ad libitum. This study was performed in accordance with the Basel Declaration guidelines on the care and use of laboratory animals. Rats were maintained with standard treatment and care, in accordance with the UK Animals (Scientific Procedures) Act 1986. Rats were sacrificed by CO_2_ asphyxiation, in accordance with Code of Practice for the Humane Killing of Animals, under Schedule 1 of the Animals (Scientific Procedures) Act, 1986. Animal studies are reported in compliance with the ARRIVE guidelines (*https://www.nc3rs.org.uk/arrive-guidelines*, [63]).

CB-MSCs were isolated from the long bones, as previously described [12]. CB-MSCs were subsequently expanded at 37 °C/5% CO_2_ in normal glucose (5.5 mM) or high glucose (25 mM) complete culture medium (CCM), consisting of αMEM with ribonucleosides and deoxyribonuclosides, supplemented with 20% heat-inactivated foetal bovine serum (FBS, ThermoFisher Scientific, Paisley, UK), 1% antibiotics-antimycotics and 100 μM L-ascorbic acid 2-phosphate (both Sigma-Aldrich, Poole, UK); for ~ 350 days in culture [12].

### Assessment of population doubling levels

Upon reaching 70–80% confluence, CB-MSCs in normal (5.5 mM) and high (25 mM) glucose were treated with StemPro**®** Accutase**®** (ThermoFisher Scientific) and PD rates calculated from cell counts throughout the ~ 350 days in culture, as previously described [12, 39]. Cumulative PDs were subsequently plotted against time in culture, with the onset of cellular senescence confirmed when CB-MSCs underwent < 0.5PDs/week [12, 39].

### Cell morphology analysis

CB-MSC morphologies at PD15, PD50, PD100 and PD200 under normal (5.5 mM) and high (25 mM) glucose conditions, were examined using FITC-phalloidin staining and fluorescence microscopy (Olympus Provis AX70 Microscope, Olympus UK Ltd., Southend-on-Sea, UK), as previously described [12]. CB-MSC surface areas were quantified by ImageJ Software (NIH Software, Version 1.49).

### Telomere length determination

Following DNA purification, CB-MSC telomere length assessments at PD15, PD50, PD100 and PD200 in normal (5.5 mM) and high (25 mM) glucose, were performed using the TeloTAGGG Telomere Restriction Fragment Length (TRF) Assay Kit (Roche, Welwyn Garden City, UK). Mean telomere lengths were subsequently calculated from Southern blot images using ImageJ® Software, as previously described [12, 39].

### Senescence associated-β-galactosidase staining

CB-MSCs senescence at PD15, PD50, PD100 and PD200 under normal (5.5 mM) and high (25 mM) glucose conditions, was assessed by the presence of senescence associated (SA)-β-galactosidase staining, using a Senescence Cells Histochemical Staining Kit (Sigma-Aldrich), as previously described [12, 39].

### Quantitative real-time PCR

For the determination of cellular senescence marker (p53, p21^waf1^ and p16^INK4a^) expression by CB-MSCs at PD15, PD50, PD100 and PD200 in normal (5.5 mM) and high (25 mM) glucose using quantitative real-time polymerase chain reaction (Q-PCR), total RNA extraction and cDNA generation were performed, as previously described [12, 39]. For each Q-PCR reaction, 2 μL of 3 μM primers (forward and reverse sequences detailed in Table 1; all Primer Design Ltd., Southampton, UK), were mixed with 10 μL of 2x Precision FAST Q-PCR Master Mix (Primer Design); and 1 μL nuclease-free water. 5 μL cDNA (1 μg isolated cDNA, diluted 1,10 in nuclease-free water), was applied in triplicate into 96-well Q-PCR plates (Primer Design), followed by 15 μL of pre-prepared Master Mix. Q-PCR reactions were run on a QuantStudio™ 6 Flex Real-Time PCR System Machine (ThermoFisher Scientific), using QuantStudio™ Real-Time PCR Software (v1.0). Reaction conditions had an initial denaturing step of 95 °C (20 s), followed by 40 cycles at 95 °C (1 min), 1 annealing cycle of 62 C (20 s), denaturation at 95 °C (15 s), dissociation at 60 °C (1 min); and denaturation at 95 °C (15 s). GAPDH served as an internal control for data normalisation. Gene expression quantification was calculated using the ΔCt method, through gene expression as a percentage of the GAPDH housekeeping gene [12].

**Table 1:** Primers sequences for RT-PCR and Q-PCR analysis

Gene	Primer Sequence	Length (bp)	Application
p53	F: ACAGCGTGGTGGTACCGTAT R: GGAGCTGTTGCACATGTACT	83	Q-PCR
p21^waf1^	F: TCTTGCACTCTGGTGTCTCA R: GGGCTTTCTCTTGCAGAAG	147	Q-PCR
p16^INK4A^	F: TGCAGATAGACTAGCCAGGGC R: CTCGCAGTTCGAATCTGCAC	184	Q-PCR
GAPDH	F: GCAAGAGAGAGGCCCTCAG R: TGTGAGGGAGATGCTCAGTG	74	Q-PCR
rTERT	F: GCTCCGGTTACACAGCAGCCCCTGGCA R: GGTCCAGAGCACGCACACGCAGCACGA	752	RT-PCR
TR	F: CTCCGCCCGCTGTTTTTCTCGCTGACT R: GCCACCGAACTCAGGGACCAGTTCCGT	300	RT-PCR
CD73	F: TCCCGCGGCTGCTACGGCACCCAAGTG R: ACCTTGGTGAAGAGCCGGGCCACGCCG	204	RT-PCR
CD90	F: CCTGACCCGAGAGAAGAA R: TGAAGTTGGCTAGAGTAAGGA	125	RT-PCR
CD105	F: CGGTCTCCAGCTGCGGTGGTGGGCTCC R: CACTGCCACCACGGGCTCCCGCTTGCT	896	RT-PCR
CD34	F: GTCACACTGCCTACTACTTC R:TCCTCGGATTCCTGAACAT	210	RT-PCR
CD45	F: AGCAATACCAGTTCCTCTATGA R: TCCGTCCACTTCGTTATGA	113	RT-PCR
CD106	F: TCCACACTGACGCTGAGCCCTGTGGGTG R: CTCCGGCATCCTGCAGCTGTGCCTTGCG	898	RT-PCR
CD146	F: GCAGCGCCACGGGTGTGCCAGGAGAGG R: CCCCACTGTGGTGCTTCTGGGCGGGCT	900	RT-PCR
Nanog	F: GGGGATTCCTCGCCGATGCCTGCCGTT R: GGGATACTCCACCGGCGCTGAGCCCTT	477	RT-PCR
Oct4	F: GCCCACCTTCCCCATGGCTGGACACCT R: GCAGGGCCTCGAAGCGGCAGATGGTTG	563	RT-PCR
Slug	F: CACTCCCCTCTGCCCAGCGGCCTTTCT R: GGCATGGGGGTCTGAAAGCTTGGGCTG	171	RT-PCR
Snail	F: GCGAGCTGCAGGACGCGTGTGTGGAGT R: CGGCAAAGGCACGGTTGCAGTGGGAGC	597	RT-PCR
β-actin	F: TGAAGATCAAGATCATTGCTCCTCC R: CTAGAAGCATTTGCGGTGGACGATG	155	RT-PCR
Osterix	F: GCTTTTCTGTGGCAAGAGGTTC R: CTGATGTTTGCTCAAGTGGTCG	136	Q-PCR
OCN	F: AAGCCCAGCGACTCTGAGTCT R: CCGGAGTCTATTCACCACCTTACT	75	Q-PCR
Adiponectin	F: GAATCATTATGACGGCAGCAC R: CTTGGAGCCAGACTTGGTCTC	224	Q-PCR
PPARγ	F: GGAAGCCCTTTGGTGACTTTATGG R: GCAGCAGGTTGTCTTGGATGTC	174	Q-PCR
β-actin	F: TGAAGATCAAGATCATTGCTCCTCC R:CTAGAAGCATTTGCGGTGGACGATG	108	Q-PCR

### Reverse transcription PCR

Reverse transcription PCR was employed for the determination of senescence (rTERT and TR) and stem cell (CD73, CD90, CD105, CD34, CD45, CD106, CD146, Nanog, Oct4, Slug and Snail) marker expression; by CB-MSCs at PD15, PD50, PD100 and PD200 under normal (5.5 mM) and high (25 mM) glucose conditions. Total RNA extraction, cDNA generation and PCR reactions were performed as previously described [12, 39], using primer sequences described in Table 1, with β-actin serving as the reference housekeeping gene. PCR products and 100 bp DNA ladders (Promega, Southampton, UK) were separated on 2% agarose gels in 1x Tris-acetate-EDTA buffer. Images were captured under UV light and analysed as previously described [12, 39].

### Colony forming efficiencies

CB-MSCs colony forming efficiencies at PD15, PD50, PD100 and PD200 in normal (5.5 mM) and high (25 mM) glucose were determined as previously described, with colonies defined as containing > 32 cells [12, 64].

### Assessment of osteogenic differentiation

The osteogenic differentiation capabilities of CB-MSCs at PD15, PD50, PD100 and PD200 under normal (5.5 mM) and high (25 mM) glucose conditions, were determined following CB-MSC culture in normal (5.5 mM) and high (25 mM) glucose, osteogenic medium (consisting of αMEM with ribonucleosides and deoxyribonuclosides, supplemented with 10% FBS, 1% antibiotics-antimycotics, 100 μM L-ascorbic acid 2-phosphate, 10 nM dexamethasone and 100 mM β-glycerophosphate; all Sigma-Aldrich) [12, 39]. Negative controls were also established, with CB-MSCs remaining in non-osteogenic basal medium in normal (5.5 mM) and high (25 mM) glucose conditions. Cells were maintained at 37 °C/5% CO_2_ for 28 days, with culture medium changed every 2 days.

Total RNA was extracted and mRNA expression of osteogenic markers, osterix and OCN (both Primer Design, Table 1); examined on day 2 and day 7 (osterix) and day 21 and day 28 (OCN) by Q-PCR, as above. At day 28, cells were also assessed for mineral calcium deposition assessed by Alizarin red S staining [12, 39]. The extent of mineral formation was also quantified, as previously described [65]. Absorbance values were measured using a SPECTROstar Omega Microplate Spectrophotometer (BMG Labtech, Aylesbury, UK), at 405 nm.

### Assessment of adipogenic differentiation

The adipogenic differentiation capabilities of CB-MSCs at PD15, PD50, PD100 and under normal (5.5 mM) and high (25 mM) glucose conditions, were determined following CB-MSC culture in normal (5.5 mM) and high (25 mM) glucose, adipogenic induction medium (AIM, consisting of αMEM with ribonucleosides and deoxyribonuclosides, supplemented with 10% FBS, 1% antibiotics-antimycotics, 100 μM L-ascorbic acid 2-phosphate, 1 μM dexamethasone, 100 μM 3-isobutyl-1-methyxanthine and 100 μM indomethacin; all Sigma-Aldrich) [12]. Negative controls were also established, with CB-MSCs remaining in non-adipogenic basal medium in normal (5.5 mM) and high (25 mM) glucose conditions. Cells were maintained at 37 °C/5% CO_2_ for 6 days, with culture medium changed every 2 days. AIM was subsequently replaced by normal (5.5 mM) and high (25 mM) glucose, adipogenic maintenance medium (AMM, consisting of αMEM with ribonucleosides and deoxyribonuclosides, supplemented with 10% FBS, 1% antibiotics-antimycotics, 100 μM L-ascorbic acid 2-phosphate and 10 μg/mL insulin; all Sigma-Aldrich) [12]. Cells were maintained at 37 °C/5% CO_2_ for 2 days and re-cultured in AIM with normal (5.5 mM) or high (25 mM) glucose, until day 15, followed by repeated culture in AMM until day 17 and culture in AIM until day 21.

Total RNA was extracted and mRNA expression of adipogenic markers, adiponectin and PPARγ (both Primer Design, Table 1); examined on day 0 and 21 by Q-PCR, as described above. At day 21, cells were also assessed for intracellular lipid-rich vacuole accumulation. Cells were transferred to glass chamber slides (BD Biosciences, Oxford, UK), fixed with 4% paraformaldehyde solution (Santa Cruz, Dallas, USA) and stained with HCS LipidTOX™ Green Neutral Lipid Stain (1:200 in phosphate buffered saline, PBS; ThermoFisher Scientific), according to manufacturer’s instructions. Cells were washed in PBS (× 3), slide chambers removed and stained with DAPI Vectashield Hard-Set Mounting Medium (Vector Laboratories, Peterborough, UK). Coverslips were added and images captured by fluorescence microscopy, as above.

### Statistical analysis

Data were expressed as mean ± standard error of mean (SEM). Data were statistically compared using Analysis of Variance (ANOVA), with post-hoc Tukey test. Statistical significance was considered at *p* < 0.05.

## Data Availability

All datasets generated or analysed during this study are included in this article. The datasets supporting the conclusions of this article are available in the figshare repository (*https://doi.org/10.6084/m9.figshare.8288663.v1*).

## Abbreviations

AGEs: Advanced glycation end products
AIM: Adipogenic induction medium
AMM: Adipogenic maintenance medium
ANOVA: Analysis of Variance
BM-MSCs: Bone marrow-derived, mesenchymal stromal cells
BMPs: Bone morphogenic proteins
CB-MSCs: Compact bone-derived, mesenchymal stromal cells
CCM: Complete culture medium
CFEs: Colony forming efficiencies
dNTPs: deoxynucleotide triphosphates
ECM: Extracellular matrix
FBS: Foetal bovine serum
FITC: Fluorescein isothiocyanate
HSEs: Hematopoietic stem cells
IGF: Insulin growth factor
M-MLV: Moloney murine leukaemia virus
MSCs: Mesenchymal stromal cells
OCN: Osteocalcin
PBS: Phosphate buffered saline
PDs: Peroxisomal proliferator-activated receptor-γ
Q-PCR: Quantitative real-time polymerase chain reaction
rTERT: rat telomerase
SA: Senescence associated
SEM: Standard error of mean
T2DM: Type 2 diabetes mellitus
TA: Transit amplifying
TBS: Tris-buffered saline
TGF-β_1_: Transforming growth factor-β_1_
TR: Telomerase RNA
TRF: Telomere Restriction Fragment Length
